# Quantitative Phosphoproteomics Reveals the Requirement of DYRK1-Mediated Phosphorylation of Ion Transport- and Cell Junction-Related Proteins for Notochord Lumenogenesis in Ascidian

**DOI:** 10.3390/cells12060921

**Published:** 2023-03-16

**Authors:** Zhuqing Wang, Xiuke Ouyang, Zicheng Tan, Likun Yang, Bo Dong

**Affiliations:** 1 Fang Zongxi Center, MoE Key Laboratory of Marine Genetics and Breeding, College of Marine Life Sciences, Ocean University of China, Qingdao 266003, China; 2 Laoshan Laboratory, Qingdao 266237, China; 3 MoE Key Laboratory of Evolution & Marine Biodiversity, Ocean University of China, Qingdao 266003, China

**Keywords:** *Ciona* notochord, phosphoproteomics, proteomics, lumen expansion, vesicle trafficking, transmembrane transport, tight junction

## Abstract

The dual-specificity tyrosine phosphorylation-regulated kinase (DYRK1) phosphorylates diverse substrates involved in various cellular processes. Here, we found that blocking the kinase activity of DYRK1 inhibited notochord development and lumenogenesis in ascidian *Ciona savignyi*. By performing phosphoproteomics in conjunction with notochord-specific proteomics, we identified 1065 notochord-specific phosphoproteins that were present during lumen inflation, of which 428 differentially phosphorylated proteins (DPPs) were identified after inhibition of DYRK1 kinase activity. These DPPs were significantly enriched in metal ion transmembrane transporter activity, protein transport and localization, and tight junction. We next analyzed the downregulated phosphoproteins and focused on those belonging to the solute carrier (SLC), Ras-related protein (RAB), and tight junction protein (TJP) families. In vivo phospho-deficient study showed that alanine mutations on the phosphosites of these proteins resulted in defects of lumenogenesis during *Ciona* notochord development, demonstrating the crucial roles of phosphorylation of transmembrane transport-, vesicle trafficking-, and tight junction-related proteins in lumen formation. Overall, our study provides a valuable data resource for investigating notochord lumenogenesis and uncovers the molecular mechanisms of DYRK1-mediated notochord development and lumen inflation.

## 1. Introduction

The dual-specificity tyrosine phosphorylation-regulated protein kinases (DYRKs) are evolutionarily conserved protein kinases, of which DYRK1 is the one that has been most extensively studied. DYRK1 proteins from different species exert their conserved functions by sharing a protein kinase domain and some highly conserved motifs (such as ATP anchor, activation loop, and catalytic loop) [1]. The ATP anchor is responsible for covering and anchoring the nontransferable phosphates and the adenine ring of ATP. DYRK1 achieves full enzyme activity by auto-phosphorylating the second tyrosine of the activation loop. The catalytic loop is responsible for transferring the γ-phosphate of the ATP to the substrate [2]. DYRK1 interacts with and phosphorylates a broad range of substrates involved in various cellular processes, including cell cycle control, synaptic function, vertebrate development, and neurodegeneration [2,3]. DYRK1 is an important upstream regulator of the cytoskeleton, which inhibits the actin polymerization by phosphorylating neural Wiskott–Aldrich syndrome protein (N-WASP) at three sites [4] and regulates microtubule dynamics by directly phosphorylating β-tubulin [5]. The hyperphosphorylation of the tau protein mediated by DYRK1 promotes the dissociation of tau from the microtubules, thereby destructing the stabilization of microtubules [6]. In addition, the cytoskeleton plays an indispensable role in vesicle-dependent cargo transport. DYRK1-mediated phosphorylation of endocytic proteins is required for dynamics of endocytic complex assembly and disassembly in neuron development [7,8,9]. These previous studies suggest the roles of DYRK1 on cytoskeleton regulation and vesicle trafficking.

Notochord development of marine urochordate ascidian undergoes complex cellular processes, including convergent extension, notochord elongation, and extracellular lumenogenesis, which have been extensively characterized [10,11,12]. After convergent extension, notochord forms a single-diameter cell column and then elongates approximately two-fold along the anterior–posterior (A-P) axis driven by a cytokinesis-like actomyosin ring [13]. Meantime, the tail also undergoes extension. In addition to the contribution of epidermal cell proliferation, tail extension is mainly dependent on notochord elongation [14]. With lumenogenesis, the center of each lateral domain of notochord cells differentiates and establishes apical polarity, where the polarity proteins and ion transporters localize, enabling each notochord cell to consequently develop two apical/luminal domains [15,16,17]. Subsequently, the lumen continues to expand, which is coordinated by luminal membrane biogenesis, lateral domain regression, and lumen pocket inflation [10,12,18,19]. Recently, the exploration and discoveries of the formation of ascidian notochord and lumenogenesis have provided an ideal model for studying the mechanism of chordate notochord development and biological lumen formation [11]; however, the molecular regulatory mechanism underlying these processes remain largely unknown.

During notochord lumenogenesis, transmembrane ion transporter (SLC26aα) was proven to be vital for controlling lumen expansion and lumen size, through regulation of the luminal cross-membrane osmotic gradient [15,20]. Our previous work also revealed the essential role of intracellular vesicle trafficking on lumenogenesis in *Ciona* notochord [18]. Recently, 4D proteomics of *Ciona* notochord tissue during lumen formation has demonstrated that lumen expansion requires the orchestration of several biological processes, including ion transport, vesicle trafficking, and extracellular matrix (ECM) deposition [21]. Meanwhile, the report revealed the requirement of homeostasis of intracellular vesicle trafficking and ECM glycosylation modification for notochord lumen development and inflation by in vivo experiments [21]. Moreover, the tight junction apparatus at the apical/lateral junction ring, which circumscribes the lumen, undergoes highly dynamic remodeling to regulate lumen expansion [22]. However, how these biological processes cooperate during lumenogenesis remains poorly understood, thus urging further studies to investigate and reveal the molecular orchestration essential for tubulogenesis.

Here, we found that the kinase activity of DYRK1 contributed to *Ciona* notochord development and lumen inflation by a chemical inhibitor experiment. Phosphoproteomics was performed to identify the phosphoproteins involved in notochord lumenogenesis. In conjunction with our notochord proteomic data, we identified 1065 notochord-specific phosphoproteins with 428 differentially phosphorylated proteins (DPPs) potentially regulated by DYRK1. Moreover, we demonstrated the vital functions of the proteins related to vesicle transport, ion transmembrane transport, and tight junctions during notochord development and lumenogenesis by the analysis of downregulated phosphoproteins and loss-of-function experiments in vivo. The present study identified notochord-specific phosphoproteins involved in lumenogenesis and revealed the requirement of DYRK1-mediated ion transport and cell junction for notochord tubulogenesis.

## 2. Materials and Methods

### 2.1. Experimental Animal and Sample Preparation

The adult *Ciona savignyi* were collected from Qingdao coast (Shandong, China) during their breeding season (usually in the summer) and were maintained in a seawater aquarium. The adult animals (5–10 individuals) were dissected, and the mature eggs and sperm were collected and fertilized in the filtered artificial seawater. After fertilization, the eggs were dechorionated according to previous description [23]. The fertilized and dechorionated embryos were cultured at 16 °C in an incubator without illumination. To inhibit the phosphokinase activity of DYRK1, embryos (~100) were treated with 40 μM AZ191 (Sigma Aldrich, St. Louis, MO, USA), dissolved in dimethyl sulfoxide (DMSO) (Solarbio, Beijing, China), at 16 h post fertilization (hpf) and were collected at 20, 22, and 24 hpf, respectively. During this period, the embryos underwent notochord cell elongation and lumenogenesis [11,13,16]. Embryos (~100) treated with DMSO were set as the negative control. All treatments were repeated at least three times.

### 2.2. Phosphoproteome and Bioinformatic Analysis

The total protein of AZ191- or DMSO-treated embryos (~2000 for each treatment) was extracted and cleaved by trypsin, followed by phosphopeptide enrichment High-Select™ TiO_2_ Phosphopeptide Enrichment kit [24] (Thermo Fisher Scientific, Waltham, MA, USA). The phosphopeptides were identified using timsTOF Pro (Bruker, Ettlingen, Germany) mass spectrometry (MS) in Bioprofile biotech Co., Ltd., (Shanghai, China). The MS data were analyzed for data interpretation and protein identification against the *C. savignyi* genome annotation information re-sequenced by our lab (unpublished). Some information about this genome was described in our recent report [21]. The MS spectra were searched using MSFragger (version 2.4, https://msfragger.nesvilab.org (accessed on 1 October 2022)) and FragPipe (version 13.1, https://github.com/Nesvilab/FragPipe (accessed on 1 October 2022)) with mass calibration and parameter optimization enabled. When searching the database, the Max Missed Cleavages were set to 2. Tryptic cleavage specificity was applied, along with variable methionine oxidation (M), variable protein N-terminal acetylation, variable phosphorylation on serine (S), threonine (T), and tyrosine (Y), and fixed carbamidomethyl cysteine modifications. The allowed peptide length and mass ranges were 6–50 residues and 500–5000 Da, respectively. PeptideProphet and ProteinProphet in Philosopher (version 2.2.0; https://philosopher.nesvilab.org/ (accessed on 5 October 2022)) were used to filter all phosphosite, peptide-spectrum matches (PSMs), peptides, and proteins with <1% false discovery rate. Entries from decoy proteins were removed. Label-free quantification (LFQ) analysis was performed with IonQuant (version 1.1.0, https://ionquant.nesvilab.org (accessed on 5 October 2022)). Site quantitation analysis was filtered only for those phosphorylation sites that were confidently localized (class I, localization probability > 0.75).

To annotate the sequences, information was extracted from UniProtKB/Swiss-Prot (https://www.expasy.org/resources/uniprotkb-swiss-prot (accessed on 10 October 2022)), Kyoto Encyclopedia of Genes and Genomes (KEGG, https://www.genome.jp/kegg (accessed on 10 October 2022)), and Gene Ontology (GO, http://geneontology.org (accessed on 10 October 2022)). To detect the potential enrichment of GO terms in the differentially phosphorylated protein list, GO enrichment analysis was conducted. Proteins were classified by GO annotation into three categories: biological process (BP), cellular compartment (CC), and molecular function (MF). For GO and KEGG enrichment analyses, a Fisher’s exact test was employed to test the enrichment of the differentially phosphorylated protein against all identified proteins. The GO with a corrected *p* value < 0.05 is considered as significant.

### 2.3. Plasmids Construction and Electroporation

To obtain cDNA fragments of SLC12A6, SLC28A3, Rab8b, Rab10, and TJP2, PCR was performed with Phanta Max Super-Fidelity DNA Polymerase (Vazyme, Nanjing, China) and respective primers (Supplementary Table S5) using *C. savignyi* cDNA as a template. The cDNA of embryos during different developmental stages was extracted and synthesized by PrimeScript^™^ II 1st Strand cDNA Synthesis Kit (Takara, Beijing, China). After purification with the GeneJET Gel Extraction Kit (Thermo Fisher Scientific), the PCR products were ligated to BamHI-digested reconstructive pEGFP-N1 vector (containing a *Ciona* Brachyury promoter) using the ClonExpress MultiS One Step Cloning Kit (Vazyme). The mutants were generated by PCR amplification with the specific primers that included base substitutions at the corresponding phosphosites (Supplementary Table S5), using the respective plasmids as templates. The identified and conserved phosphosites of Rab8b-S116, Rab10-T174, SLC12A6-S975 and -S984, SLC8A3-S110, and TJP2-S116 and -S370 were substituted with neutral amino acid alanine (A), resulting in phosphorylation-deficient mutants. All the primers in this study were synthesized by Sangon Biotech (Shanghai, China). The constructed plasmids were amplified and extracted with Genopure Plasmid Maxi Kit (Roche, Basel, Switzerland).

Electroporation was performed as previously described [25] with several modifications. After fertilization and dechorionation, 300 μL of eggs with fresh seawater were mixed with 80 μL of plasmids (40 μg) and 420 μL of 0.77 M D-mannitol (Sigma Aldrich) and electroporated using a Gene Pulser Xcell System (Bio-Rad, Hercules, CA, USA) in 0.4 cm cuvettes. The exponential pulse protocol was used with the following conditions: voltage, 50 V; capacitance, 2000 μF; and resistance, ∞. After electroporation, the fertilized eggs were washed and cultured at 16 °C.

### 2.4. Immunostaining

The embryos with treatment were collected at 20, 22, and 24 hpf into 1.5 mL tubes, respectively. The collected embryos were fixed with 4% paraformaldehyde (Sigma Aldrich) at room temperature for 2 h and were washed thrice with phosphate-buffered saline (PBS) containing 0.1% Triton X-100 (Solarbio) (PBST) for 20 min each time. To observe the lumen, fixed embryos were stained with 1/200 Alexa Fluor™ 488 Phalloidin (Thermo Fisher Scientific) in PBST overnight at 4 °C. Embryos were then washed thrice with PBST and mounted on the slides with Vectashield mounting medium with 4′,6-diamidino-2-phenylindole (DAPI) (Vector Laboratories, Newark, NJ, USA). Images were taken using Zeiss LSM900 Confocal Microscope at 63X lens (Zeiss, Oberkochen, Germany).

### 2.5. Statistical Analysis

The tail length, notochord anteroposterior (A-P) cell length, and the lumen diameter were measured using an image processing package Fiji (version 2.9.0, National Institutes of Health, Bethesda, MD, USA) [26]. All graphs in this study were produced using GraphPad Prism (Version 9.3.1, GraphPad Software, San Diego, CA, USA) or gglot2 in R (version 4.2.0). Significance differences were calculated using one-way ANOVA in Microsoft Excel 2022. *p* < 0.05 was considered statistically significant.

## 3. Results

### 3.1. Kinase Activity of DYRK1 Is Required for Ciona Notochord Development and Lumen Inflation

**Figure 1:**
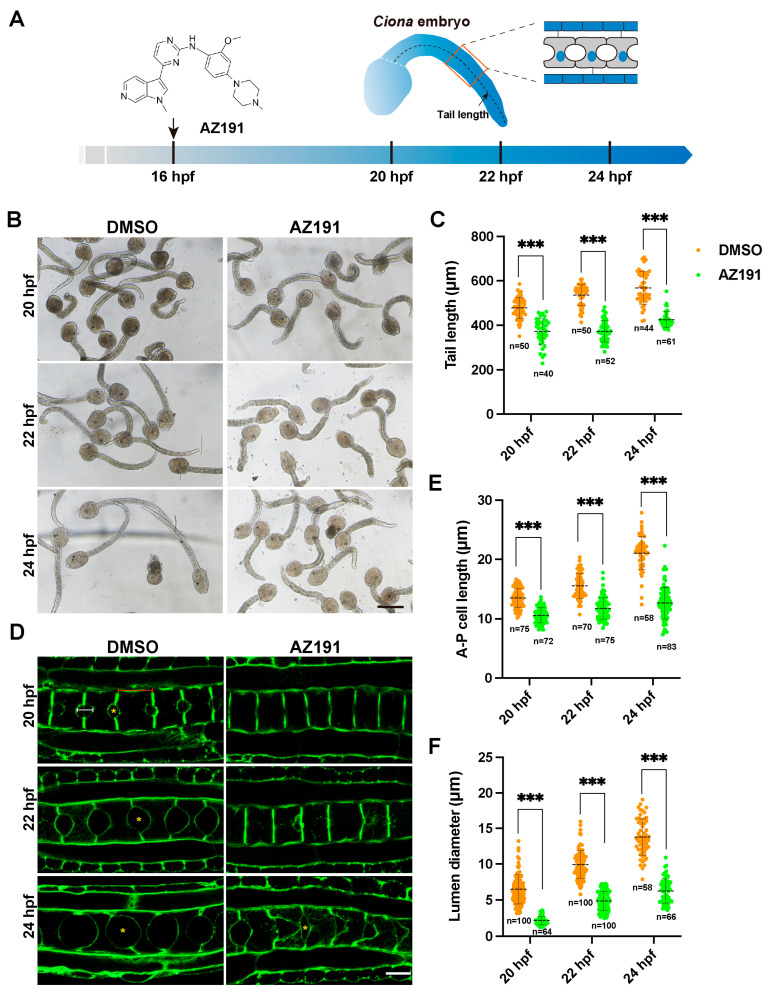
DYRK1 inhibitor AZ191 delayed the tail elongation, notochord cell elongation, and lumen inflation of *Ciona* tailbud larvae. (**A**) Schematic diagram of *Ciona* tailbud larvae treated with AZ191 during notochord elongation and lumen inflation. The black curve indicates the tail length, and the tail is locally magnified in the red frame. (**B**) The bright-field images showing the *Ciona* tailbud larvae treated with DMSO or AZ191. Bar: 200 μm. (**C**) The tail length of *Ciona* tailbud larvae treated with AZ191 was significantly shorter than that treated with DMSO. “n” represents the number of embryos used in statistical analysis. (**D**) The confocal images showing the notochord cells of *Ciona* tailbud larvae treated with DMSO or AZ191 during 20–24 hpf. Bar: 10 μm. The yellow asterisks indicate the lumen. The red and white lines represent the notochord A-P cell length and lumen diameter, respectively. (**E**) The A-P cell length of notochord treated with AZ191 was significantly shorter than the control. The “n” represents the number of notochord cells used in statistical analysis. (**F**) The A-P lumen diameter of notochord treated with AZ191 was significantly shorter than that treated with DMSO. The “n” represents the number of lumens used in the statistical analysis. Data are presented as mean ± SD. Asterisks represent statistical significance: *** *p* < 0.001 (one-way ANOVA).

Development of the *Ciona* notochord involves cell elongation and lumen formation from 17 to 23 hpf [11] (Figure 1A). Our previous transcriptomic study has revealed that DYRK1 is upregulated at 18–24 hpf (hour post fertilization) of *Ciona* embryo, during which notochord elongated and lumen formed and expanded [27], suggesting the potential role of DYRK1 on notochord development and lumenogenesis. To explore the role of DYRK1, we used AZ191, a potent selective inhibitor of DYRK1 [28], to block the kinase activity of DYRK1 of *Ciona* tailbud larva at 16 hpf, before completion of notochord elongation and lumen formation. Then, we collected the AZ191-treated embryos to investigate the tail length and lumen size (Figure 1A). The tail length of AZ191-treated embryos was apparently shorter than that of controls (DMSO-treated embryos) (Figure 1B). We next measured tail length of treated embryos at 20, 22, 24 hpf, respectively (Figure 1A, Supplementary Table S1), and the statistical results showed that the tail length of AZ191-treated embryos was shorter than the control, with significant differences at all three time points (Figure 1C). Moreover, we examined whether the process of notochord cell elongation could be affected by AZ191. We stained the treated embryos with phalloidin to mark the cell boundaries after collection and measured the anterior–posterior axis (A-P) length of notochord cells. The results showed that the A-P cell length of control was increased approximately 1.5-fold from 20 to 24 hpf. However, there was little change in the cell length of AZ191-treated embryos (Figure 1D). The quantitative statistical results showed that the A-P cell length of AZ191-treated embryos was significantly shorter than the control at all three time points (Figure 1E, Supplementary Table S2).

Furthermore, to investigate the effects of AZ191 on lumenogenesis, we measured the lumen A-P diameters (Figure 1D, Supplementary Table S3). The results showed that the lumen formed at 20 hpf, and lumen diameters increased by approximately twice from 20 to 24 hpf upon DMSO treatment (Figure 1D,F). However, the notochord lumen of AZ191-treated embryos formed without expansion at 20 hpf, and the lumen diameters were significantly smaller than those of the control groups at all three stages (Figure 1D,F). At 24 hpf, the lumen diameter of DMSO-treated embryos was already twice than those of AZ191-treated (Figure 1F). These results showed that the lumen expansion rate was remarkably reduced by AZ191. Together, the kinase activity of DYRK1 was required for *Ciona* notochord elongation and lumen expansion.

### 3.2. Phosphoproteomic Analysis of Embryos with DYRK1 Inhibition

**Figure 2:**
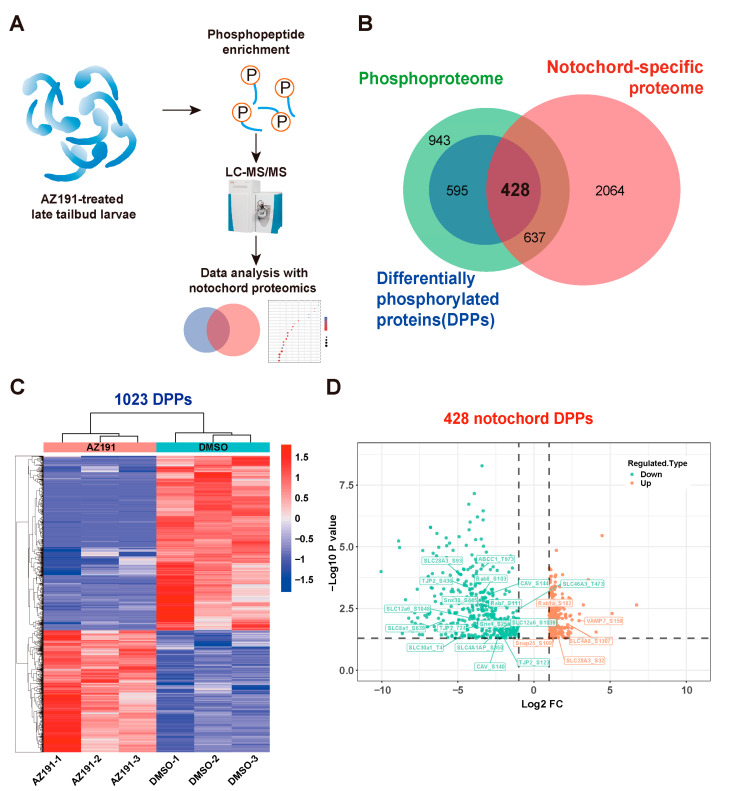
Phosphoproteomic analysis of AZ191-treated late tailbud larvae of *Ciona*. (**A**) Workflow of AZ191-treated late tailbud larvae collection, phosphopeptide enrichment, mass spectrometry, and phosphoproteomic analysis. (**B**) Venn diagram showing overlapped phosphoproteins between the phosphoproteomics and notochord proteomics. (**C**) Heatmap of DPPs in the inhibitor AZ191-treated group versus the control group. Rows represent the abundance ratio values of proteins, and columns represent individual samples (three biological replicates for each group). The colored bars show the row-standardized signal values: the red representing high and the blue representing low phosphorylation levels. (**D**) Volcano plot of the phosphorylation levels of 699 phosphosites of 428 DPPs specifically expressed in notochord tissue. The orange points indicate upregulated phosphoproteins, while green points indicate downregulated phosphoproteins. *p* value < 0.05.

To identify the specific phosphoproteins mediated by DYRK1 and associated with notochord elongation and lumen inflation, we performed phosphoproteomics with AZ191-treated tailbud larvae and data analysis (Figure 2A). Phosphoproteomics identified a total of 2603 phosphorylated proteins that possessed 5988 phosphosites. There were 1556 significant differentially phosphorylated residues corresponding to 1023 (39.3% of total phosphorylated proteins) differentially phosphorylated proteins (DPPs) following the AZ191 treatment (Figure 2B). The identified significant DPPs mediated by DYRK1 are shown by a heatmap (Figure 2C): the phosphorylation levels of 638 phosphosites (490 phosphoproteins) were upregulated, and those of 918 (533 phosphoproteins) were downregulated. Given that AZ191 is an inhibitor of DYRK1, the sites with upregulated phosphorylation levels may not be directly caused by DYRK1 inhibition.

In addition, the relative standard deviation (RSD) of protein phosphorylation intensity between the repeated samples was less than 0.2, showing great biological repeatability (Supplementary Figure S1A). We normalized the phosphoproteomics data of each treated embryo, and the intensity was normalized between 15 and 19 (Supplementary Figure S1B). Most of the phosphorylation modification sites occurred in serine (83.78%), followed by threonine (13.51%) and tyrosine (2.70%) (Supplementary Figure S1C). To reveal specific motif(s) recognized by DYRK1 in *Ciona*, peptides with downregulated phosphorylation level were analyzed using MeMe software. Eleven putative phosphorylation motifs were identified, which included nine serine motifs and two threonine motifs (Supplementary Figure S1D,F). In these two types of motifs, proline was shown to be the mostly presented residue at the position (P + 1) after the phosphorylation site (P), with frequencies of 44.49% in the serine motif and 56.57% in the threonine motif (Supplementary Figure S1D–G). This suggests that the proline at P + 1 position may play a key role in phosphosite recognition on substrates of DYRK1.

### 3.3. DYRK1-Mediated Multi-Processes Participated in Ciona Tailbud Larva Notochord Development

Since the inhibition of DYRK1 affected the notochord development and lumenogenesis, we further identified the phosphoproteins specifically expressed in notochord tissue. To this aim, we performed Venn analysis combining phosphoproteomics with notochord proteomics and then identified 1065 notochord-specific phosphoproteins (Figure 2B, Supplementary Table S4). The GO enrichment analysis showed that these phosphoproteins were enriched in: (1) transition metal ion binding, transferase activity, transferring glycosyl group, sequence-specific DNA binding, tubulin binding, molecular carrier activity and ribosome binding of MF; (2) intrinsic and integral component of membrane and AP-type membrane coat adaptor complex, and proton-transporting ATP synthase complex of CC; (3) macromolecule localization, cellular protein localization, regulation of cellular protein metabolic process, and regulation of translation of BP (Supplementary Figure S2A). The KEGG enrichment showed that these notochord-specific phosphoproteins were enriched in multiple neurodegenerations, lysosomes, the apelin signaling pathway, tight junctions, endocytosis, the HIF-1 signaling pathway, and the N-glycan biosynthesis pathway (Supplementary Figure S2B). These enrichment results suggest that multiple binding function, cellular protein localization, and metabolic processes might be regulated by post-translational modification (phosphorylation).

**Figure 3:**
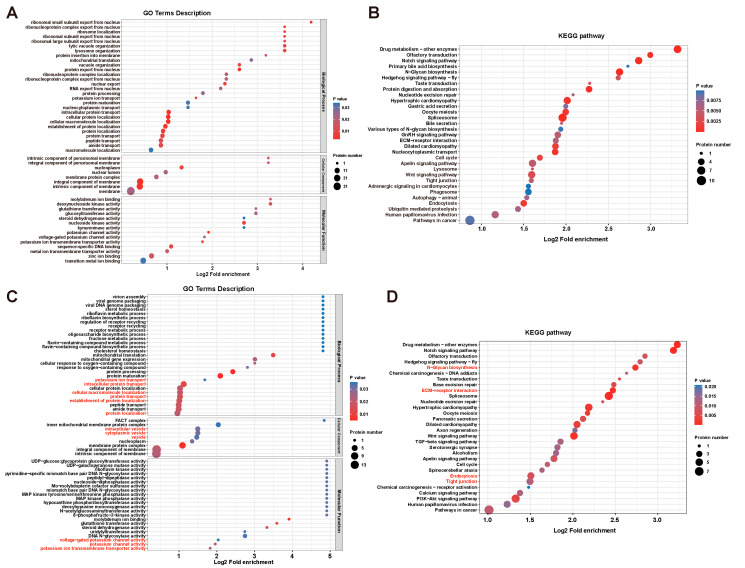
Identification and analysis of DPPs specifically expressed in notochord tissue. (**A**) GO enrichment analysis of the DPPs in notochord. (**B**) KEGG pathway enrichment analysis of the DPPs in notochord. (**C**) GO enrichment analysis of the notochord-specific phosphoproteins with significant downregulated phosphorylation level. (**D**) KEGG pathway enrichment analysis of the downregulated phosphoproteins in notochord. The main enriched items involved in lumenogenesis are highlighted in red.

To particularly investigate the action of DYRK1 on notochord-specific phosphoproteins, we found 428 significant DPPs in notochord (41.8% of total DPPs) caused by DYRK1 inhibition (Figure 2B). Furthermore, the volcano plot showed that the phosphorylation levels of 416 phosphosites were downregulated significantly, and those of 283 phosphosites were upregulated significantly (Figure 2D). GO enrichment analysis showed that these notochord-specific DPPs were enriched in transition metal ion binding, zinc ion binding, metal ion transmembrane transporter activity, sequence-specific DNA binding, voltage-gated potassium channel activity, and glucosyltransferase activity of MF; membrane and membrane protein complex of CC; protein transport, localization, and potassium ion transport of BP (Figure 3A). Moreover, these significant DPPs were enriched in ubiquitin-mediated proteolysis, endocytosis, tight junction, Wnt signaling pathway, apelin signaling pathway, ECM-receptor interaction, GnRH signaling pathway, various types of N-glycan biosynthesis, spliceosome, and Notch signaling pathway through KEGG enrichment (Figure 3B). Therefore, the biological processes and pathways mentioned above potentially participated in *Ciona* notochord development.

Furthermore, since AZ191 inhibits the kinase activity of DYRK1, downregulated phosphoproteins were more likely to be directly regulated by DYRK1. The identification of downregulated phosphoproteins after AZ191 treatment will be helpful in further elucidating the molecular mechanisms underlying notochord development and lumenogenesis. Therefore, we screened the significantly downregulated phosphoproteins in notochord and performed enrichment analysis. We found that these downregulated phosphoproteins were enriched in potassium ion transmembrane transport activity, potassium channel activity, and voltage-potassium channel activity of MF; intrinsic and integral component of membrane and vesicle of CC; protein localization, establishment of protein localization, protein transport, cellular macromolecule localization, and potassium ion transport of BP (Figure 3C). In addition, KEGG showed the enrichment of endocytosis, tight junction, and N-glycan biosynthesis (Figure 3D).

### 3.4. DYRK1-Mediated Transmembrane Transport and Tight Junction Were Essential for Notochord Lumen Inflation

**Figure 4:**
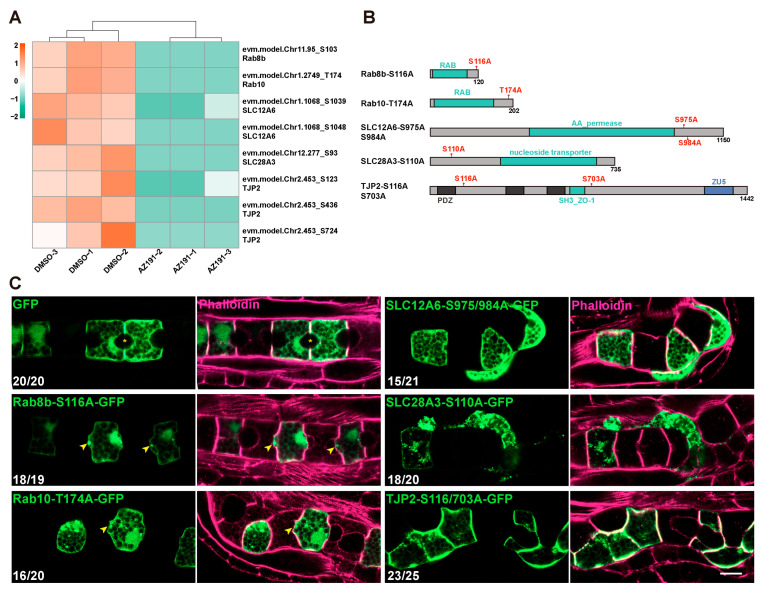
The analysis and in vivo phospho-deficient experiments of the transport-, ion transmembrane transport-, and tight junction-related proteins. (**A**) The phosphorylation level change of Rab8b, Rab10, SLC12A6, SLC28A3, and TJP2 after treatment with AZ191. Colors denote the phosphorylation levels (orange to green; high to low). (**B**) The schematic diagram of the construction of phospho-deficient mutants. (**C**) Confocal images showing the phenotypes of mutants of Rab8b, Rab10, SLC12A6, SLC28A3, and TJP2. The asterisk indicates lumen between two adjacent notochord cells. The arrows indicate the cell bulging. Bars: 10 μm. “n/n” represents the ratio of normal embryos/the total embryos in the GFP group or the ratio of lumen-defective embryos/the total embryos in phospho-deficient groups.

Given the potential roles of protein transport and localization, ion transmembrane transport activity, and tight junction during notochord lumen formation, we further showed the phosphorylation level of the proteins related to these three functions. These proteins included SLC28A3, SLC12a6, SLC46A3, SLC30a1, and SLC4A1AP of solute carriers (SLC) family and Rab8, Rab7, and Rab1a of Ras-related proteins (Figure 2D). We focused on several related proteins, including Rab8b, Rab10, SLC12A6, SLC28A3, and tight junction protein (TJP2), and found that the phosphorylation levels of all their phosphosites were downregulated significantly after AZ191 treatment (Figure 2D and Figure 4A).

To functionally verify the roles of the potential DYRK1-targeted phosphoproteins mentioned above, we identified the conserved phosphosites of these proteins through alignment with the sequences of other species and then constructed the phosphorylation-deficient mutants by substituting these sites with neutral amino acid alanine (A) (Figure 4B). We electroporated them into *Ciona* embryos to show their expression in notochord cells using a notochord-specific promoter *Brachyury.* The late tailbud embryos were fixed and stained with phalloidin to mark the cell boundaries. In the control, a spheroidal lumen was normally formed between the two notochord cells expressing GFP (Figure 4C). However, the notochord cells expressing both the Rab8b-S116A-GFP and Rab10-T174A-GFP bulged toward the region where the lumen was expected to form. The AP-cell length of mutant cells was shorter than that of the control (Figure 4C). Moreover, both the SLC12A6 and SLC8A3 without phosphorylation (SLC12A6-S975/984A-GFP and SLC8A3-S110A-GFP) resulted in abnormal notochord cell arrangement and lumenogenesis (Figure 4C). In addition, non-phosphorylated TJP2-S116/370A-GFP led to significantly disordered notochord cell arrangement (Figure 4C).

Taken together, the phospho-deficient experiments in vivo indicated that phosphorylation of the Rabs, SLC, and tight junction proteins was vital for the cell shape and arrangement of notochord cells and lumenogenesis.

## 4. Discussion

The evolutionally conserved protein kinase DYRK family can interact and phosphorylate various substrates involved in different cellular processes, including cell proliferation, cell differentiation, cell survival, cell migration, and so on [2]. In this study, the preference for proline residues at the P + 1 position of *Ciona* DYRK1 (Supplementary Figure S1D–G) is consistent with the previous notion that DYRK1 was classified as proline-directed kinases, thus indicating that the phosphosite recognition mechanism of *Ciona* DYRK1 is highly conserved.

Furthermore, we found that AZ191 significantly delayed tail extension and lumen expansion, suggesting that kinase activity of DYRK1 was critical for *Ciona* notochord development and lumenogenesis. Length extension of *Ciona* embryos was mainly dependent on notochord cell elongation along the A-P axis [13]. Notochord cell elongation is driven by a cytokinesis-like actomyosin ring at the equator of each cell, which is composed of a phosphorylated myosin regulatory light chain, α-actinin, cofilin, tropomyosin, and talin [13,29,30]. We demonstrated that DYRK1 played an essential role in the regulation of larval notochord development and lumenogenesis by treating embryos with chemical inhibitor AZ191. The elongation of notochord cells was significantly delayed by AZ191 treatment (Figure 1E), leading to a short-tail phenotype in swimming larvae. We found that the phosphorylated levels of α-actinin (evm.model.Chr2.450_T455) and talin (evm.model.Chr1.329_S955, T1261), key components for the actomyosin ring, were regulated by DYRK1 (Supplementary Table S4). Therefore, DYRK1, by exerting its kinase activity, may phosphorylate these components to ensure their stability, thereby regulating notochord cell elongation. However, this still needs further investigation.

Notochord lumenogenesis requires the orchestration of several biological processes, including polarity establishment [17,22,30,31], vesicle-mediated protein transport [18], ion transmembrane transport [15], and ECM secretion and deposition [27]. In this study, by performing phosphoproteomics in conjunction with notochord-specific proteomics, we identified DPPs related to protein transport and localization, ion transmembrane transport, and tight junctions (Figure 2D), which were regulated by DYRK1 during lumenogenesis. Rab8b is a GTPase essential for vesicle transport of proteins to the plasma membrane prior to secretion [32,33,34,35,36]. Rab10 is involved in a myriad of functions in the polarized transport of proteins from the Golgi to the plasma membrane [37], and in exocytosis [38] and endosomal sorting [39] in polarized cells. Accumulated findings have suggested that the dynamic control of the Rab function is more regulated by phosphorylation [40,41]. We have found that non-phosphorylated Rab8b or Rab10 resulted in cell bulging, probably due to protein retention in the cells with disorder of polarized transport (Figure 4C). In addition, the shorter A-P cell length also appeared in Rab8b or Rab10 mutant cells, indicating the contribution of membrane growth to cell elongation [29]. Besides Rab8b and Rab10, several intracellular trafficking-related proteins, including Rab1a, Rab7, SNX30, SNAP25, SEC61b, and VAMP7, were identified to be regulated by DYRK1 in the notochord (Figure 2D). These investigations clearly suggest that DYRK1-mediated intracellular trafficking is essential for notochord lumen formation.

Our results have shown that many solute carrier family proteins (SLCs) are regulated by DYRK1, including SLC12A6, SLC28A3, SLC46A3, and SLC4A8 (Figure 2D). Furthermore, we demonstrated the destructive effect of phosphorylation-deficient mutants of SLC12A6 and SLC28A3 on notochord cell arrangement and lumenogenesis. These results suggest that DYRK1-mediated phosphorylation of ion transmembrane transporters was also required for notochord development and lumen formation. As an electroneutral potassium-chloride cotransporter, SLC12A6 regulates intracellular chloride concentrations and contributes to cell volume homeostasis [42,43]. SLC28A3 is a concentrative nucleoside transporter protein and regulates multiple cellular processes, such as vascular tone [44]. Meanwhile, SLC26aα, another member of the SLC family, has been identified as an anion transporter, regulating the osmotic pressure inside and outside the *Ciona* notochord lumen and thus participating in the regulation of lumen size [15]. Therefore, it is logical to conclude that SLC-mediated transmembrane transport has crucial functions on notochord development and lumenogenesis. In addition to the SLC family, we found that tight junction protein TJP2 (also named ZO-1), a tight junction component involved in lumen initiation and expansion [17], has three phosphosites (evm.model.Chr2.453_S123, S436, S724) that are regulated by DYRK1 (Figure 4A). Non-phosphorylated TJP2-S116-GFP lost its polarized membrane localization [17] and localized all over the plasma membrane instead, which may be the reason for the disordered notochord cell arrangement (Figure 4C). The result demonstrated that the phosphorylated TJP2 was essential for notochord cell arrangement. In addition, caveolin with multiple phosphosites (evm.model.Chr2.763_S131, S138, S140, S144) regulated by DYRK1 is responsible for apical membrane curvature during *Ciona* lumen expansion [18]. These investigations suggested an upstream regulation function of *Ciona* DYRK1 on notochord development.

Human DYRK1 is highly expressed in the nervous system and has received much attention due to special localization on the Down syndrome critical region (DSCR) of chromosome 21 [45]. Some studies from vertebrates confirmed that DYRK1 is vital to the regulation of nervous system development and function [46,47]. The KEGG enrichment of *Ciona* DYRK1 in neurodegenerative disease-related pathways, including Alzheimer’s disease, Huntington’s disease, and Parkinson’s disease (Supplementary Figure S2B), suggests that *Ciona* DYRK1 also participated in the regulation of nervous system development in ascidian larvae. Therefore, this function of DYRK1 may be conserved in chordates. Further studies are required to delineate the role of DYRK1 in *Ciona* nervous development.

Our phosphoproteome revealed 1023 DPPs after AZ191 treatment, representing 39.3% of the identified phosphoproteins (Figure 2B). The presence of a high proportion of DYRK1-related phosphoproteins might be explained by the fact that phosphoproteomics was performed on treated embryos at a specific developmental stage when DYRK1 was highly expressed. In addition, a large proportion (41.8%) of the 1023 DPPs was expressed in notochord tissue (Figure 2B), further indicating the significance of DYRK1 in notochord development.

Overall, our phosphoproteomics and functional verification revealed that DYRK1-mediated phosphorylation of the proteins related to protein transport, ion transmembrane transport, and tight junctions was critical for *Ciona* notochord development and lumenogenesis. This study provides insights into uncovering the molecular mechanisms underlying chordate notochord development.

## Data Availability

The phosphoproteomics data have been deposited to the ProteomeXchange Consortium via the iProX partner repository with the dataset identifier PXD039234. The mass spectrometry proteomics data of notochord have been deposited into the ProteomeXchange Consortium via the PRIDE partner repository with the dataset identifier PXD037089.

## Supplementary Materials

The following supporting information can be downloaded at: https://www.mdpi.com/article/10.3390/cells12060921/s1, Figure S1: Analysis of phosphoproteomics; Figure S2: GO and KEGG pathway enrichment analysis of notochord phosphoproteins; Table S1: The tail length of AZ191-treated *Ciona* embryos; Table S2: The A-P cell length of AZ191-treated *Ciona* notochord cells; Table S3: The lumen diameter of AZ191-treated *Ciona* notochord; Table S4: Phosphoproteins in the notochord; Table S5: Primers used in this study.
