# Supplementary material for: Quantitative Phosphoproteomics Reveals the Requirement of DYRK1-Mediated Phosphorylation of Ion Transport- and Cell Junction-Related Proteins for Notochord Lumenogenesis in Ascidian
Source: Cells. 2023 Mar 16;12(6):921. doi: 10.3390/cells12060921 (PMC10047359; doi:10.3390/cells12060921)
Supplement: Supplementary file 1 [file cells-12-00921-s001.zip › cells-2131060 supplementary conversion.pdf]

Supplementary Material

Supplementary Figures and Tables

1.1 Supplementary Figures

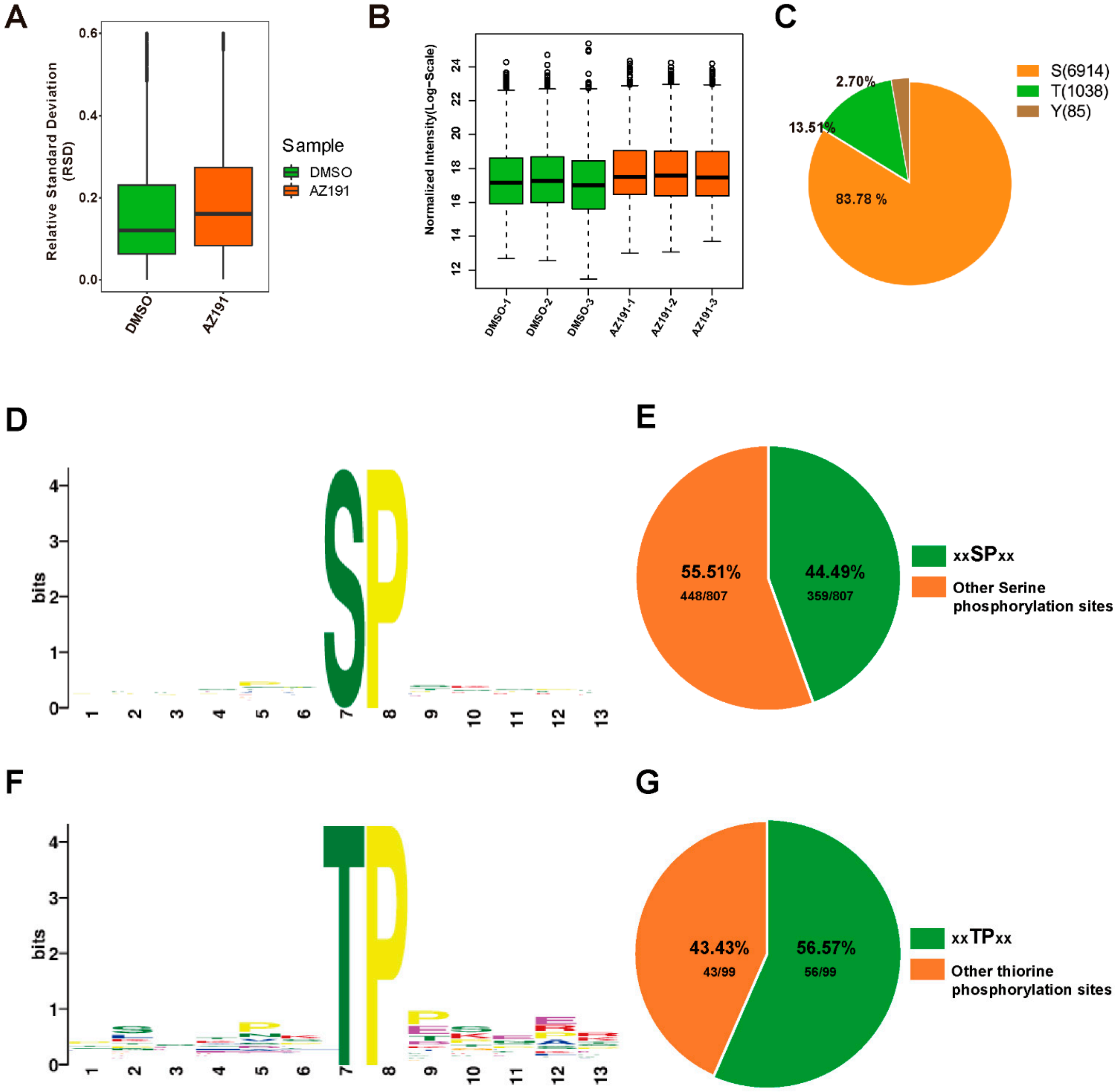

Supplementary Figure S1. Analysis of phosphoproteomics. (A) The RSD plot showing great biological repeatability of each duplicate samples. (B) Normalized intensity of duplicate samples. (C) Distribution of phospho-Ser/Thr/Tyr in the identified phosphoproteins. (D and F) The majority motifs in significantly down-regulated phosphorylation sites. The height of the residues represents their

frequency of occurrence at the respective positions. (E) Distribution of xxSPxx motif in all serine motifs. (G) Distribution of xxTPxx motif in all threonine motifs. The position of other amino acids in the motif indicates the position relative to the phosphorylation site (P). In serine motifs, the P+1 position is proline (approximately 44.49%). In threonine motifs, the P+1 position is proline approximately 56.57%.

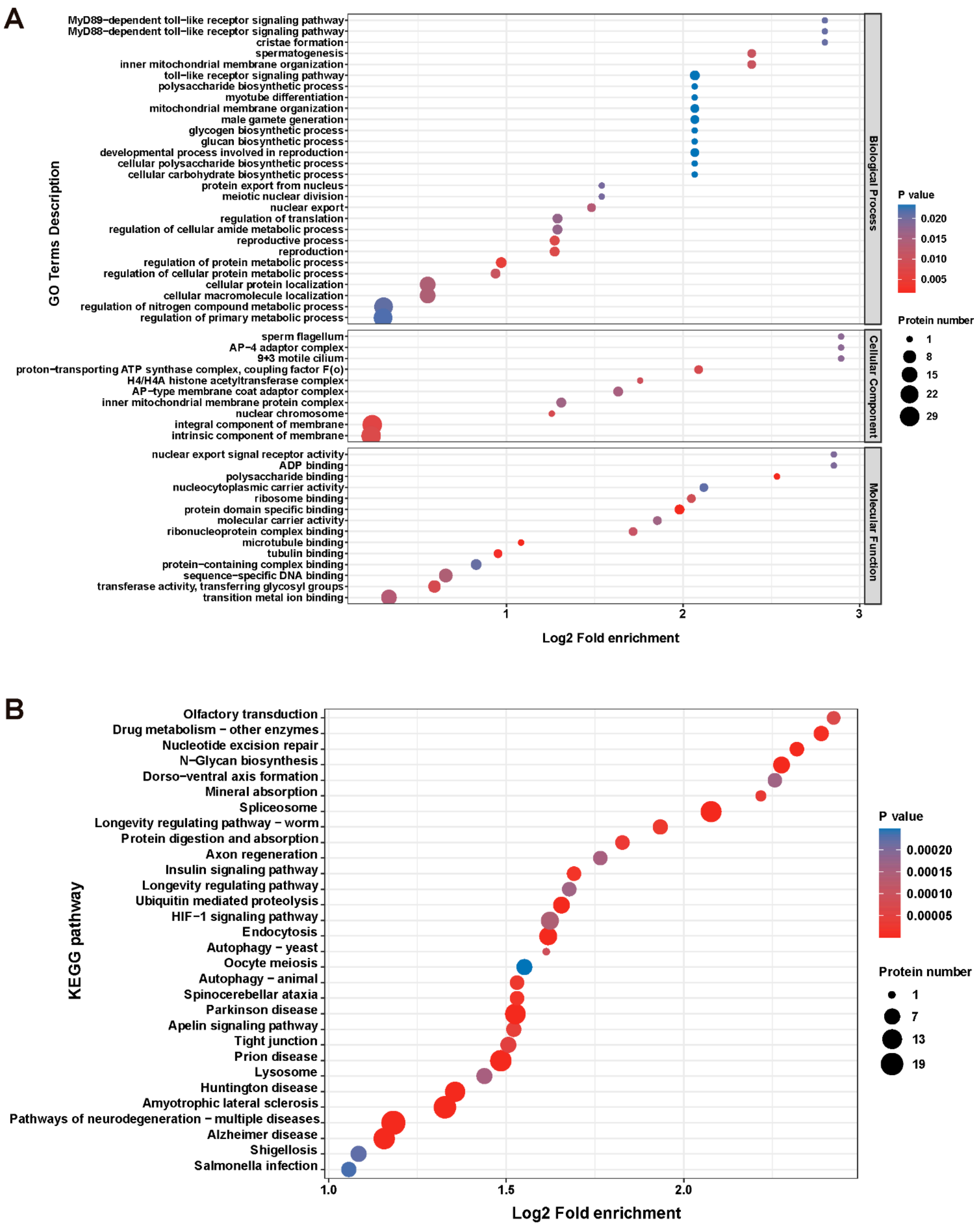

Supplementary Figure S2. GO and KEGG pathway enrichment analysis of notochord phosphoproteins. (A) GO enrichment analysis of notochord phosphoproteins. (B) KEGG pathway enrichment analysis of notochord phosphoproteins.

1.2 Supplementary Tables (in the separated files)

Supplementary Table S1. The tail length of AZ191-treated *Ciona* embryos.

Supplementary Table S2. The A-P cell length of AZ191-treated *Ciona* notochord cells.

Supplementary Table S3. The lumen diameter of AZ191-treated *Ciona* notochord.

Supplementary Table S4. Phosphoproteins in notochord.

Supplementary Table S5. Primers used in this study.
